# Retraction to “Study to compare the effect of casirivimab and imdevimab, remdesivir, and favipiravir on progression and multi-organ function of hospitalized COVID-19 patients”

**DOI:** 10.1515/med-2023-0853

**Published:** 2023-11-10

**Authors:** Sahar K. Hegazy, Samar Tharwat, Ahmed H. Hassan

**Affiliations:** Clinical Pharmacy Department, Faculty of Pharmacy, Tanta University, Tanta, PC: 31511, Egypt; Rheumatology and Immunology Department, Faculty of Medicine, Mansoura University, Mansoura, PC: 35511, Egypt; Clinical Pharmacy Department, Mansoura University Hospital, Mansoura, PC: 35511, Egypt

The publisher would like to inform that the article, Hegazy S., Tharwat S, Hassan A. Study to compare the effect of casirivimab and imdevimab, remdesivir, and favipiravir on progression and multi-organ function of hospitalized COVID-19 patients Open Medicine 2023;18(1):20230768, DOI: 10.1515/med-2023-0768, has been retracted.

Editorial Office decided to retract this article due to significant overlap with the other papers published by the same authors in *Journal of Clinical Virology Plus*: doi.org/10.1016/j.jcvp.2023.100151.

